# Finger Tapping as a Biomarker to Classify Cognitive Status in 80+-Year-Olds

**DOI:** 10.3390/jpm12020286

**Published:** 2022-02-15

**Authors:** Dieter F. Kutz, Stephanie Fröhlich, Julian Rudisch, Katrin Müller, Claudia Voelcker-Rehage

**Affiliations:** 1 Department of Neuromotor Behavior and Exercise, Institute of Sport and Exercise Sciences, University of Muenster, 48149 Münster, Germany; stephanie.froehlich@uni-muenster.de (S.F.); julian.rudisch@uni-muenster.de (J.R.); claudia.voelcker-rehage@uni-muenster.de (C.V.-R.); 2 Institute of Human Movement Science and Health, Faculty of Behavioural and Social Sciences, Chemnitz University of Technology, 09126 Chemnitz, Germany; katrin.mueller@hsw.tu-chemnitz.de

**Keywords:** aging, cerebellum, classification, cognitive decline, diadochokinesia, motor control, sensory motor performance, time perception

## Abstract

This study examined the association between finger tapping and cognitive function in a group of 225 elderly participants (116 males; age 79–92 years; M = 82.5; SD = 2.4). Finger tapping was assessed in two conditions: self-selected pace and fast pace. Based on cognitive assessments, including the MoCA and CERA-NP test battery, participants were classified as cognitively healthy individuals (CHI), participants with mild cognitive impairments (MCI), and those with possible MCI (pMCI). Results of the analyses show significant differences between groups, sex and the group × sex interaction in four parameters for the self-selected pace condition and eight parameters for the fast pace condition. These parameters were used for classification by means of linear discriminant analysis (LDA). The first LDA component showed significant differences between CHI and pMCI and between CHI and MCI. Furthermore, the second LDA component showed significant differences between CHI and pMCI as well as between pMCI and MCI. Nevertheless, the algorithm correctly classified only 50% of participants, regardless of group, suggesting that tapping parameters are only partially useful for classification in early stages of dementia. We discuss these findings in terms of the diadochokinetic nature of finger tapping as associated with the age-related degeneration of cortical and subcortical motor areas.

## 1. Introduction

As societies age, more and more people become affected with dementia [1]. In Germany, according to the German Alzheimer Society e.V. [2], the number of people with dementia will have risen to three million by 2050. In addition to the personal cost, the disease causes substantial economic and social burdens [3]. However, these burdens can be alleviated by early diagnosis of dementia and its pre-stages, as such early detection can allow for more sustainable disease management and optimal health care for affected individuals [1]. It is therefore important to identify people with pre-dementia (e.g., persons with mild cognitive impairments, or MCI) early enough so they can start programs that will help them maintain their personal lifestyle and that will continuously assess the course of the dementia as it progresses.

In many therapeutic areas, diseases and treatments are evaluated using patient-reported outcome (PRO) measures (subjective measures), collected, for example, via questionnaires [4]. However, several barriers exist for using PRO measures in cognitive impairment. For example, disease-related disorders can impair memory and cause people to lose insight into how their disease is progressing [4,5]. In these cases, one must rely on the reports of clinicians or information from personal contacts, such as family members [4,6]. However, the accuracy of the information provided by family members may be suboptimal, as biases may interfere or caregivers may lack knowledge regarding the disease symptoms [4,7]. Therefore, the validity of PRO measurements is limited. Furthermore, the sensitivity of current PRO measures for patients with mild cognitive impaired individuals (MCI) and Alzheimer’s disease (AD) patients in the prodromal stage is limited, as they are not specifically designed for these milder conditions [4]. A combination of several neuropsychological tests (e.g., MoCA [8] and CERAD-NP [9]) may improve classification (e.g., [10,11]) and additionally identify a transitional stage between cognitively healthy individuals (CHI) and MCI, possible MCI (pMCI) as recently defined as individuals with some signs of cognitive impairment [1,10].

AD and cognitively healthy individuals have been shown to differ in performance of movement tasks (e.g., finger tapping [12]). Therefore, objective measurements, e.g., by technical systems that measure simple movements, are an alternative to PRO measurements because they are easy to use and inexpensive. For this purpose, researchers use computer-aided measuring systems that measure parameters of the movement by means of a keyboard [13], force sensors [12] or light beams [14]. With these types of devices, studies have shown differences in finger tapping tasks between age-matched healthy subjects and people with AD [12,13,14], MCI [13,14] and Parkinson’s disease [14] in a mean age range of 71–82 years. These differences are mainly related to a slowing of the tapping rhythm and an increase in touch duration as well an increase in the variability of these parameters [12,13,14]. Such a study on the finger tapping behavior of group pMCI has not been previously conducted. In general, it should be noted that tapping is a diadochokinetic movement consisting of flexion followed by extension of the fingers. The timing of the change in movement is controlled by proprioceptive signals that are triggered when the force sensor is touched [15,16,17]. The tapping task therefore tests the ability to plan and execute rhythmically oppositely oriented movements. It is shown that the selected tap pace has an influence on the execution [13].

Therefore, in this study we aimed to use tapping parameters to distinguish between participants over 80 years old who were either cognitively healthy individuals (CHI), mild cognitive impaired individuals (MCI) or had possible MCI (pMCI), in two different conditions: as consistently as possible at a self-selected pace or as fast as possible without considering consistency (fast pace). We expected that in addition to reproducing known differences in tapping parameter between CHI and MCI groups [13,14], we would also find differences between pMCI subjects and the other groups. In addition, a recent study has shown for this study group that sex has an effect on force control [18]. It was therefore expected that sex differences in finger tapping parameters would be found. Based on these differences, we then developed a classifier to determine whether a subject belongs to a group.

## 2. Materials and Methods

This study is part of the SENDA study (Sensor-based systems for early detection of dementia, registered in the German Clinical Trials Register under DRKS00013167), which was conducted at Chemnitz University of Technology, Germany. The detailed study protocol was published earlier [1]. Only information relevant to the current research question is described here.

### 2.1. Participants

The SENDA study was advertised by local general practitioners and in newspapers. In total, 244 participants (123 males; age 79–93 years; M = 82.5; SD = 2.5) took part in the study and were recruited from January 2018 to March 2020. Study participation required walking ability, sufficient German language skills, residence in or around Chemnitz, Germany, and a self-organized means of travel to and from the laboratory. Volunteers were excluded before testing if any of the following criteria applied: (1) acute psychological disorder; (2) diagnosis of any neurocognitive or neurological disorder; (3) past traumatic head injury; (4) substance abuse; (5) participation in other clinical studies; (6) a physician-directed ban from physical activities; (7) severe restrictions due to cardiovascular, pulmonary, or orthopedic diseases; or (8) failure to reach the minimum required score of 19 during screening with the Montreal Cognitive Assessment [8]. Each participant signed a written informed consent form, and all study proceedings were approved by the Ethics Committee of Chemnitz University of Technology, Germany, Faculty of Behavioral and Social Sciences (V232-17-KM-SENDA-07112017, approved on 19 December 2017). Each participant received 25 EUR compensation for his or her participation at three appointments.

The analysis for this article included 225 participants who took part at the baseline measurement (T1, see [1]). Exclusion from analysis was due to (1) dropout from the study before all needed testing was completed (*n* = 14) or (2) technical issues during the recording (*n* = 5). Due to the participants’ old age, many followed a medication regimen (*n* = 211), which most often included medication for high blood pressure (*n* = 174), thrombosis prophylaxis, cholesterol reduction, stomach acid reduction or thyroid function. Demographic characteristics are reported in Table 1.

### 2.2. Neuropsychological Testing and MCI Classification

The neuropsychological testing and MCI classification are described in detail elsewhere [10]. Briefly, all participants went through an intensive neuropsychological test battery, which was carried out by trained testing staff at the university lab. The tests included the German version of the MoCA [8] and the German version of the Consortium to Establish a Registry for Alzheimer’s Disease Neuropsychological Test Battery [9, CERAD-NP]. The MoCA was used to measure global cognitive functioning and to screen for MCI. The MoCA is the second-most-utilized geriatric cognitive screening tool after the Mini-Mental State Examination (MMSE) and has superior sensitivity to mild cognitive impairments [19]. The CERAD-NP examines the cognitive domains of memory, language, executive functions and visuo-construction. MCI classification was based on the recommendations of The National Institute on Aging and the Alzheimer’s Association [20] and in accordance with the criteria proposed by [21]. Cognitive impairments were determined according to performance in MoCA (one sum score) and CERAD-NP (nine separate test scores). The following CERAD-NP scores were used: verbal fluency (number of animals named in 1 min), Boston naming test (number of objects correctly identified), phonematic fluency (number of words named with letter “S” in 1 min), constructional praxis (number of correctly copied characteristics), word list learning (number of words correctly remembered in third trial), word list recall (savings score), word list recognition (discriminability score), constructional praxis recall (savings score), and trail making test (quotient B/A). We followed a two-step procedure recommended for diagnosis of MCI in the general population, which states that, first, a screening should be used, and, second, in the case of abnormal findings, in-depth cognitive testing should follow [22]. A MoCA score below 26 points and at least one CERAD-NP test performance at least 1.5 standard deviations below the normative mean (taking into consideration age, sex and education level) resulted in the classification of participants with mild cognitive impairments (MCI). Correspondingly, participants were classified as being cognitively healthy individuals (CHI) if they scored 26 or more points on the MoCA and were also within the normative range (no score below −1.5 SD) in all of the CERAD-NP tests [11]. Due to the application of the two-step process, an additional class was defined for participants who showed cognitive impairments only according to one of the two tests. They were categorized as possibly having MCI (pMCI). This group included either participants who had deficits in one specific domain of the CERAD-NP but their overall cognitive functioning was normal according to MoCA, or participants who had no strong impairment in any single domain but had small deficits in different domains adding up to a low MoCA score (<26). Although this group would be considered non-MCI according to [22], as these individuals neither showed abnormal scores in the screening (MoCA > 25) nor exhibited any cognitive impairments in in-depth clinical testing after abnormal testing, we opted to analyze this group separately to have high discriminatory power between CHI and MCI.

### 2.3. Tasks and Recording

Participants carried out three fine motor tasks [1], including (1) force modulation of a precision grip with the thumb and index finger [18]; (2) tapping with the index finger of the right hand, [based on, 12]; and (3) connecting dots on a touchscreen with a touch pen/tracing (as studied by [23]). Here we report the results of the second motor task, experiment (2).

**Figure 1:**
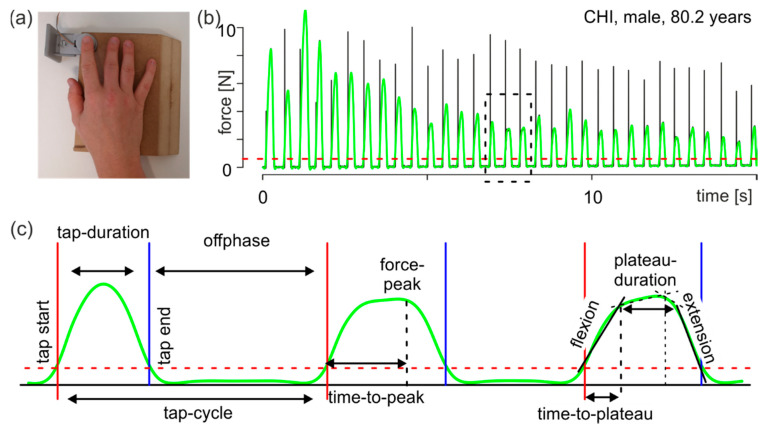
Methods. (**a**) Experimental setup. (**b**) Characteristic force curve of tapping. Black line: original recorded force curve; green line: low-pass filtered force curve; red dashed line: force threshold (see text); the dashed rectangle indicates the interval shown in c. (**c**) Example interval with three taps showing all time and force parameters (see text). Green line: filtered force curve; red vertical solid line: start point of an individual tap; blue vertical solid line: end point of an individual tap; red dashed line: force threshold; black vertical dashed line in the middle tap: time point of the force peak; black vertical dashed lines in the right tap: break points of the two-linear spline models (see text).

For the finger tapping tasks, we used one force transducer with a diameter of 29.5 mm, a depth of 8 mm, and a measurement range of 0–22.5 kg (manufacturer: Measurement Specialties Inc., Hampton, VA, USA; Model: FX-1901-0001-50 L) [18]. Signals were pre-amplified (using a customized voltage amplifier), digitally converted and sampled at a frequency of 1000 Hz using a NI-DAQ USB-6002 (National Instruments, Austin, TA, USA). The force transducer was fixed in a self-built wooden board that was placed on the table in front of the participants to prevent any movement of the transducer during the task (see Figure 1a). Experimental procedures, i.e., data acquisition, were programmed using a customized LabView 2015 (National Instruments, Austin, TA, USA) script. The task involved tapping with one’s dominant index finger on the force transducer, which participants carried out in two different conditions: as consistently as possible at a self-selected pace (cf., Figure 1b) or as fast as possible without considering consistency (fast pace). Each trial lasted 15 s. In order to reduce the influence of fatigue, the trials were carried out in blocks: the first three trials were in the self-selected pace condition and then two trials were performed for the fast pace condition. Participants received no visual feedback.

### 2.4. Data Processing, Parameter Extraction and Statistical Analyses

Data processing and parameter extraction were performed separately for each trial with a custom-made program in R ([24], ver. 3.6.3). The results were visually inspected and, when necessary, manually corrected. For determining the moment of finger contact with the force transducer, an individual threshold was calculated for each trial using a k-means algorithm with three means. The lowest mean value described the distribution of the noise of the non-contacted force transducer, the highest mean value described the distribution of the force peaks and the remaining mean value described the transition from the noise to the force peaks. The upper 95% confidence band of the first mean (the noise) was defined as the threshold. For the following analyses, the force curve was low-pass filtered using a second order Butterworth filter (cutoff frequency 100 Hz). Individual taps were identified using the filtered force curve. The tap start was defined as the moment when the force curve crossed the threshold after remaining below the threshold for the prior 100 ms. The tap end was the first moment after that when the force curve fell below the threshold (see Figure 1c). Based on the identified taps, the following parameters were extracted:

- tap duration: interval from tap start to tap end;
- tap cycle: interval from a tap start to the following tap start;
- offphase: interval from a tap end to the next tap start, namely the time when the finger is not in contact with the force transducer;
- force peak: force maximum of an individual tap; and
- time to peak: time from tap start to the moment of the force peak.

Visual inspection of the taps showed that an individual tap could be described by a trapeze (Figure 1c, right tap), having a force increase from tap start onwards (Figure 1c, flexion), reaching a plateau for several milliseconds (Figure 1c, plateau duration) and then followed by a decrease in force until tap end (Figure 1c, extension). To calculate these parameters, the tap was divided into two intervals at time to peak (first interval from tap start to time-to-peak, second from time-to-peak to tap end). For each interval, a two-linear spline model for the force curve over time was calculated [25]. From these calculations, the following parameters were extracted:

- flexion: first force slope in the first interval describing the flexion performance during tapping (Figure 1c, flexion);
- extension: second force slope of the second interval describing the extension performance during tapping (Figure 1c, extension);
- time to plateau: duration from tap start to the break point of the first interval (Figure 1c, right tap). This time describes the duration of the execution of flexion after contact of the finger with the force sensor; and
- plateau duration: duration from the first break point to the second break point (Figure 1c, right tap).

Of note, tapping is a diadochokinetic movement consisting of finger flexion followed by finger extension. The time to stop flexion and start extension is controlled by proprioceptive signals [15,16,17]. Therefore, the mean size of the time to plateau gives information on the planned movement (the shorter the time to plateau, the faster the movement), and its variability gives information on the participant’s proprioceptive control at the spinal cord level (the smaller the better).

All time values were calculated in seconds, the force peak was calculated in Newtons and flexion and extension were calculated in Newtons/second. Since the distributions of a participant’s parameters exhibited skewness and kurtosis that could not be fitted by a standard uniform procedure, each participant’s individual finger tapping behavior was characterized by two values: first, the size of a parameter using the median of the values and, second, the variability of this parameter using the inter-quartile range (iqr). These values were calculated separately for all parameters in both conditions (self-selected pace and fast pace).

Since the group data were not normally distributed, they were logarithmically transformed for statistical analysis. A mixed ANOVA with a between-subjects factor with two levels (group and sex) and a within-subjects factor (condition) was performed. ANOVA was performed using the R package ez [26]. Effect size η^2^_G_ is given to provide comparability [27]. Post-hoc comparisons were based on Fisher’s least significant difference (FLSD) when appropriate. Linear discriminant analysis (LDA) was performed using the R package MASS (version 7.3–53) based on [28]. For LDA, the logarithmized parameters were z-transformed.

## 3. Results

This study was part of the SENDA study [1] and examined the finger tapping behavior of 225 participants over 80 years old who took part at baseline measurement T1 [1]. As described above, the study participants were classified into three groups according to their cognitive performance: cognitively healthy individuals (CHI, *n* = 79), participants with possible mild cognitive impairments (pMCI, *n* = 80), and participants with mild cognitive impairments (MCI, *n* = 66). Overall, 44,813 taps were recorded: 17,724 in the self-selected pace condition and 27,089 in the fast pace condition. In the self-selected pace condition, the groups did not behave differently: CHI performed 86.5 ± 4.1 taps on average (mean ± standard error of the mean (SEM)), pMCI 74.3 ± 3.4 taps, and MCI 74.9 ± 4.6 taps. In contrast, in the fast pace condition, MCI produced 114 ± 4.2 taps, significantly less than CHI (127 ± 3.2 taps, paired *t*-test with Bonferroni’s correction, *p* = 0.019). pMCI (119 ± 2.9 taps) was not different compared with the other two groups.

### 3.1. Statistical Analyses

#### 3.1.1. ANOVA

Group means and standard error of the mean (SEM) of the logarithmized parameter for the two conditions are given in Table 2. For convenience, all group mean values of the tapping parameters have been back-transformed into the respective physical dimensions and are listed in the supplement (Supplementary Table S1). In addition, a sex-specific breakdown of the values can be found in the supplement (Supplementary Table S2 and Supplementary Table S3). ANOVA shows that a total of four (out of 18) parameters in the self-selected pace condition (Table 3) and eight (out of 18) in the fast pace condition (Table 4) differed significantly (*p* < 0.05) in group, sex, or the group × sex interaction. For post-hoc comparison of significant effects, Fisher’s least significant difference (FLSD) is given.

Mean values of the parameters (expressed as medians) were different between the groups in both conditions, i.e., the self-selected pace condition and the fast pace condition (Table 2). Post-hoc comparison of tap-cycle_median showed that inside each group, participants tapped faster in the fast pace condition than in the self-selected pace condition (FLSD (group + tapping condition) = 0.109). In contrast, group comparisons showed that only CHI differed from the other groups in the fast pace condition (FLSD (group) = 0.070, Table 4). For tap-duration_median, the post-hoc comparison showed that within each group, participants pressed the button for a shorter time during the fast pace condition than during the self-selected pace condition (FLSD (group + tapping condition) = 0.121). In contrast, in the group comparison, only MCI was significantly longer than the others in the fast pace condition (FLSD (group) = 0.070, Table 4).

In the fast pace condition, pMCI was significantly different from CHI for the parameter offphase_median and from MCI for the parameters force-peak_median, flexion_median, and extension_median. Therefore, for motor behavior, pMCI can be considered its own group between CHI and MCI. In addition, MCI differed significantly from CHI in the fast pace condition for the parameters tap-cycle_median, tap-cycle_iqr, tap-duration_median, force-peak_median, and flexion_median. In contrast, all significant variability measures showed sex differences (tap-cycle_iqr, offphase_iqr, Table 4).

Individual parameters clearly had small effect sizes, as shown by ηG^2^. The highest significant value for group was 0.0393 (plateau-duration_median at self-selected pace; Table 3), for sex it was 0.0579 (offphase_median at fast pace; Table 4), and for group × sex it was 0.046 (tap-cycle_median at fast pace; Table 4). Hence, none of the parameters alone were suitable for assigning individual participants to one of the groups. Instead, classifying participants required a combination of parameters with significant effects for group sex, or group × sex.

#### 3.1.2. Linear Discriminant Analysis

Linear discriminant analysis (LDA) is a method of finding a linear combination of features that characterizes two or more classes of parameters. The resulting combination reduces the dimensionality and is used to classify the participants. Because a recent study for this study group showed that sex has an effect on force control [18], LDA was performed not only with parameters of the effect group but also with parameters of the effect of sex and the interaction group × sex. In total, four parameters of the self-selected pace condition and eight parameters of the fast pace showed significant differences (*p* < 0.05) for the effects of group, sex or the interaction group × sex. These parameters were analyzed using the R package MASS (see [28]). The LDA showed that the parameters can be combined into two linear combinations, LDA1 and LDA2. LDA1 explains 70% of the variance and LDA2 30%. The scales of the parameters are given in Table 5. Note that the suffix _self specifies the parameter of the self-selected pace condition and _fast specifies that of the fast pace condition.

**Figure 2:**
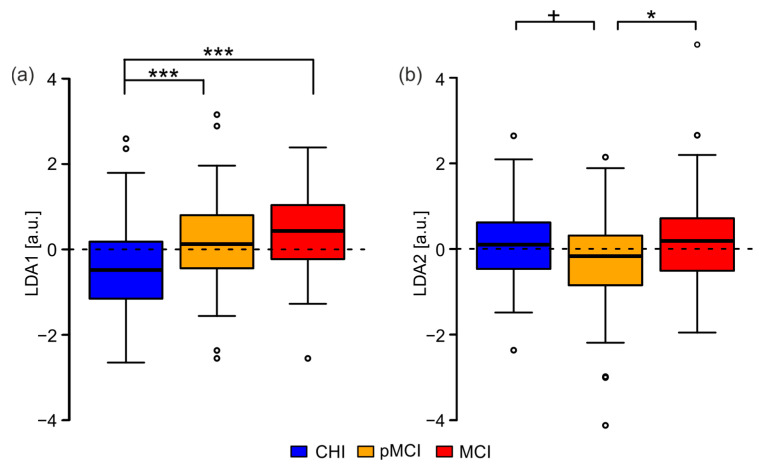
Boxplot of the LDA scales per group. (**a**) LDA1; (**b**) LDA2. CHI: cognitively healthy individuals; pMCI: participants with possible mild cognitive impairments; MCI: participants with mild cognitive impairments. ^+^ *p* < 0.1, * *p* < 0.05, *** *p* < 0.001.

The distribution of the LDA scales of each group showed a significant difference of the medians among them for LDA-1 (Figure 2a). Post-hoc tests by means of a pairwise Wilcoxon rank-sum test confirmed the group difference for LDA1 between CHI and pMCI (*p* < 0.001, Bonferroni corrected) and between CHI and MCI (*p* < 0.001, Bonferroni corrected). For LDA-2 (Figure 2b), there was a difference in medians between CHI and pMCI as well as between MCI and pMCI. The post-hoc tests for LDA2 confirmed the significant difference between pMCI and MCI (*p* < 0.05, Bonferroni corrected) and between pMCI and CHI only at a trend level (*p* < 0.1, Bonferroni corrected). The same test for CHI vs. MCI revealed that for LDA2, the two groups were not significantly different at all (*p* = 1, Bonferroni corrected).

**Figure 3:**
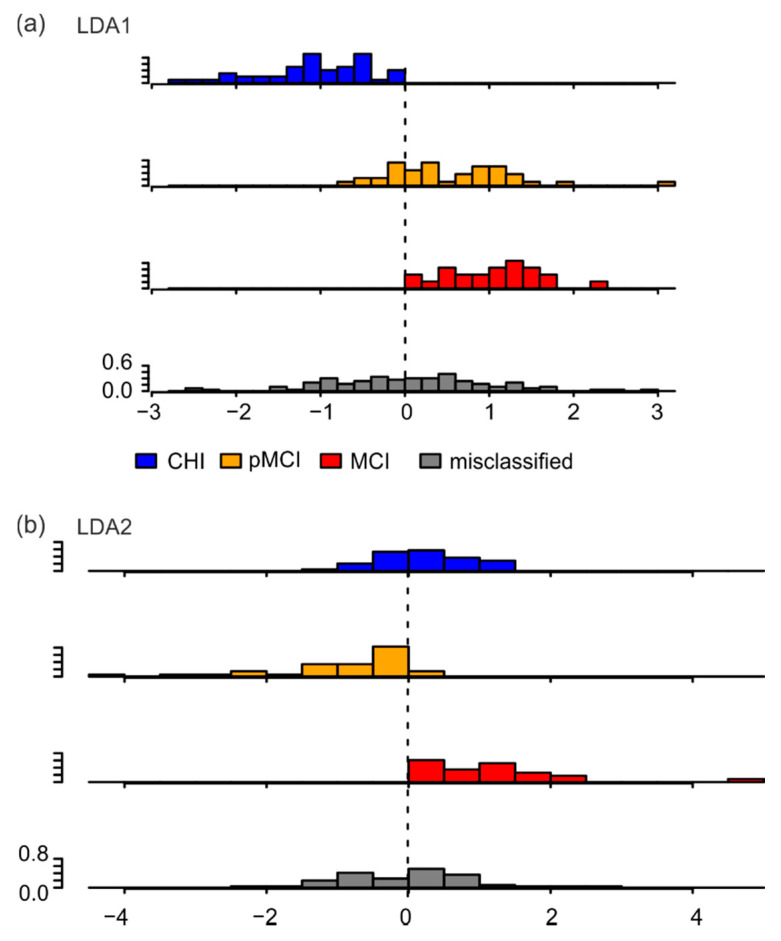
Histograms of probability densities of LDA values of correctly classified and misclassified participants. (**a**) LDA1; (**b**) LDA2. CHI: cognitively healthy individuals; pMCI: participants with possible mild cognitive impairments; MCI: participants with mild cognitive impairments.

Astonishingly, reclassifying the participants based on the linear discriminant analysis only categorized 50% of participants into the right class, with the goodness of classification decreasing from CHI (49 of 79) to pMCI (41 of 80) to MCI (23 of 65). This is better than the theoretical probability of 1/3, but 50% were still misclassified (Table 6). The sensitivity of this classification for each group was CHI = 0.62, pMCI = 0.51, and MCI = 0.35; the specificity for each group was CHI = 0.70, pMCI = 0.69, and MCI = 0.86. Upon further inspecting the distribution of LDA1 and LDA2 over all correctly classified participants and misclassified participants, we found that for LDA1, correctly classified CHI participants were indeed different on this scale relative to correctly classified pMCI and MCI participants (Figure 3a). For LDA2, the difference between correctly classified pMCI participants and correctly classified participants of the other groups was clearly visible (Figure 3b). Importantly, the LDA’s inability to properly classify participants was related to the broad distribution of misclassified participants on both LDA1 and LDA2 scales (gray histograms in Figure 3a,b).

The LDA with only parameters significant to the effect group reclassified only 47% of participants into the correct class. The distribution of LDA scales for each group showed a significant difference (*p* < 0.05) between medians only for LDA1 between CHI and pMCI and between CHI and MCI (data not shown). The histograms of the probability densities of the LDA values of correctly classified and misclassified participants mainly show a broadening of the distribution of misclassified participants (Supplement Figure S1). Overall, this indicates that finger tapping behavior was conditioned by cognitive status in only a subset of participants.

## 4. Discussion

The aim of the study was to develop a system that uses tapping parameters in a self-selected and fast tapping mode to distinguish cognitively healthy individuals (CHI) from people with possible MCI (pMCI) and people with mild cognitive impairments (MCI), specifically for individuals over 80 years old. For this purpose, the finger tapping behavior of 225 subjects over 80 years old was analyzed. ANOVA revealed differences between groups (CHI, pMCI, MCI), sexes (male, female) and their interaction (group × sex) for the self-selected pace condition (four parameters) and for the fast pace condition (eight parameters). These parameters were used for classification by means of a linear discriminant analysis (LDA). The first LDA component showed significant differences between CHI and pMCI, CHI and MCI, and pMCI and MCI. Furthermore, the second LDA component showed significant differences between CHI and pMCI and between pMCI and MCI. Nevertheless, when the algorithm was used to classify individual participants, it was correct in only 50% of cases. This shows that tapping parameters were only partially useful for classification.

Our results showed that pMCI, a group first described in the SENDA study [10], differed from both CHI and MCI. Previous studies on tapping behavior were mainly conducted with Alzheimer’s patients (e.g., [12]) or MCI patients, (e.g., the CDR. 5 group in [13]). In this study, we additionally showed that in the self-selected pace condition, not only participants with MCI but also those with pMCI had a significantly slower tapping rhythm and prolonged touch duration compared to CHI (Table 2: tap-cyle_median, tap-duration_median, and plateau-duration_median).

However, the planned goal of classifying individual participants based on tapping parameters was only partially achieved. Thus, while 49 of 79 CHI participants were correctly classified on the basis of their motor performance, 30 of these participants were classified as pMCI or MCI. Furthermore, 42 of 65 MCI patients were apparently classified as CHI or pMCI. An explanation for the misclassification might be the simplicity of the task. Previous work has shown that no age effects exist in tasks with simple planned anticipatory grasp control, such as in tapping [29]; only tasks with higher complexity, such as activities of daily living, had recognizable differences [29]. In a recent study with a subset of the subjects described here, it was shown that all participants were comparably able to perform anticipatory grip strength control regardless of group membership [18]. It can therefore be assumed that the motor requirements of the tapping task were not sufficient to reliably separate between the groups.

In addition to the experimental condition, the neurological status of the participants must also be considered. All participants reported no neurological deficits (an exclusion criterion; see Methods). However, individuals may have had different degrees of age-related degeneration and in different relevant areas of the CNS (e.g., cortex, spinal cord, basal ganglia, cerebellum). Cortical activity can be measured via resting-state electroencephalography (EEG), usually performed with eyes closed and/or eyes open [30]. It is a measure of tonic brain activity [31] and this spontaneous EEG activity is thought to account for 80% of total brain activity [30,32]. Only a small additional percentage is accounted for by engagement in a task [32]. Thus, resting-state EEG studies describe the functional state of the cortex. A recent study [10] showed that in the subjects studied, cortical activity in resting-state EEG did not differ between groups. Therefore, group differences in tapping parameters cannot be derived from cortical differences between the groups.

For Parkinson’s disease, as an example disease of the basal ganglia, it is known that patients show a faster tapping rhythm than healthy subjects [14]. In contrast, our data show that in the self-selected pace condition pMCI and MCI tapped significantly slower than CHI, and in the fast pace condition there was a significant difference between CHI and MCI for this parameter (Table 2). This is consistent with behavior shown in MCI and Alzheimer’s patients [12,13,14]. Therefore, it is reasonable to conclude that the group differences are not due to an influence of the basal ganglia.

The influence of spinal cord control can be derived from the parameter time-to-plateau (Figure 1). Tapping can be described as a diadochokinetic task. It consists of finger flexion followed by finger extension. The time to stop flexion and start extension is controlled by proprioceptive signals. The mean size of the parameter time-to-plateau gives information on the planned movement (the shorter the time to plateau, the faster the movement), and its variability gives information on the participant’s proprioceptive control at the spinal cord level (the smaller the better). The parameter time-to-plateau was determined by the current speed of the movement (Figure 1, flexion) and the sensory feedback at touch, which could lead to deceleration of the movement and onset of the reverse movement (Figure 1, extension). If sensory feedback is insufficient, the stopping of the movement is delayed and much more variable. Thus, group differences can be inferred from the variability and mean magnitude of this parameter. For the self-selected pace condition, no group differences existed in either mean magnitude (Table 2 time-to-plateau_median) or variability (Table 2 time-to-plateau_iqr). In the fast pace condition, only one significant difference was found between CHI and pMCI or MCI for the parameter time-to-plateau_median. Therefore, in the fast pace condition, CHI performed a significantly faster motor program than the other groups. Because the variability of the time-to-plateau parameter was the same between groups in both pace conditions, it can be assumed that the degree of degeneration at the spinal cord level can be considered comparable between the groups.

The cerebellum is known to be generally important for coordinating motor performance, such as diadochokinesis, and it is additionally important for associating sensory information with movements as well as for adapting movements [33]. Some studies have highlighted the cerebellum’s importance in the context of participants’ associative learning of grip forces [34,35]. For example, in precision finger tasks such as the raspberry task [36,37], half of the young participants showed a conditioned change in force at just the second presentation of the conditioning stimulus [35] and personal observation of DFK. In contrast, cerebellar patients were significantly worse than control subjects at learning the necessary association [34]. For a successful association between the conditioned stimulus and the motor action, participants needed a well-planned and controlled execution of the task [35]; in cerebellar patients, this execution was impaired [25]. It is therefore possible that restrictions in tapping behavior can be explained not only by cognitive impairments, but also by age-related decline of the cerebellum. This is accompanied by a reduced ability to associate sensory information with the necessary timing of tapping. As they are spatially separated from the regions related to manual motor performance, parts of the cerebellum are also correlated with cognitive performance [38,39,40,41]. The anterior lobe and the top of the superior posterior lobe are correlated with motor skills, and the bottom parts of the posterior superior lobe and the inferior lobe are correlated with cognition [39]. Degeneration of cerebellar regions associated with the somatomotor network is more pronounced than that of regions associated with dorsal attention, ventral attention, or frontoparietal networks [38]. Furthermore, age-related degeneration of the motor cerebellum is comparable to the degeneration found in cerebellar diseases [38]. In contrast, Alzheimer’s patients show degeneration of the cognitive part of the cerebellum without concomitant increased degeneration in the motor cerebellum [41]. Notably, the cerebellum is generally considered to be resistant to the neurotoxic effects of soluble amyloid-beta (Aβ), which is helpful in the early stages of AD [42]. However, assuming that a proportion of participants classified as MCI are in a precursor phase to AD, it is still reasonable to hypothesize that the influence of the cerebellum on tapping behavior should be considered an age-related limitation rather than an effect of the developing disease.

In conclusion, the 30 misclassified participants in the CHI group may have had more degeneration of the motor cerebellum than those correctly classified into the CHI group (*n* = 49). Indicators for this difference are the values for the tap-cycle (correctly classified CHI: −0.697 ± 0.008; misclassified CHI: −0.422 ± 0.013; mean ± SEM in log [s]) and tap-duration (correctly classified CHI: −1.825 ± 0.007; misclassified CHI: −1.448 ± 0.016; mean ± SEM in log [s]) parameters in the self-selected condition. Thus, in this condition, the correctly classified CHI showed a significantly faster tapping rhythm with a shorter tapping duration (*p* < 0.005, Bonferroni corrected, both parameters). Similarly, it can be hypothesized that the 42 misclassified MCI participants had less degeneration of the cerebellum than the correctly classified MCI patients (*n* = 23). This can be seen from the values for the tap-cycle (correctly classified MCI: −1,289 ± 0.012; misclassified MCI: −1.407 ± 0.005; mean ± SEM in log [s]) and tap-duration (correctly classified MCI: −2.017 ± 0.012; misclassified MCI: −2.213 ± 0.004; mean ± SEM in log [s]) parameters in the fast pace condition. In this condition, the correctly classified MCI showed a slower tap rhythm (*p* < 0.1, Bonferroni corrected) with a significantly longer tap duration (*p* < 0.004, Bonferroni corrected).

Overall, when investigating whether cognitive state can be assessed based on simple finger movements (such as tapping), one must also consider the possible degeneration of relevant motor systems (e.g., the cerebellum). To establish tapping as a good classifier, researchers need to perform additional motor tests to specifically determine the degeneration of the aforementioned areas and adequately assess their impact on tapping behavior.

## Figures and Tables

**Table 1:** Characteristics of the groups according to cognitive status.

	CHI	pMCI	MCI
*n* (in %)	79 (35)	80 (36)	66 (29)
m/f	35/44	43/37	38/28
Age in years	82.0 ± 0.3	82.5 ± 0.2	82.9 ± 0.3
Education in years	14.3 ± 0.4	13.9 ± 0.4	13.7 ± 0.4
MMSE (0–30)	28.3 ± 0.2	27.8 ± 0.2	27.3 ± 0.2
MoCA (0–30)	27.7 ± 0.1	25.8 ± 0.2	22.8 ± 0.2

Given are means ± SEM. CHI: cognitively healthy individuals; pMCI: possible MCI; MCI: mild cognitive impaired individuals. For details of the classification, see text. f: female, m: male; MMSE: Mini-Mental State Examination; MoCA: Montreal Cognitive Assessment.

**Table 2:** Logarithmized tapping parameters for both conditions for each group.

	Self-Selected Pace			Fast Pace		
	CHI (*n* = 79)	pMCI (*n* = 80)	MCI (*n* = 66)	CHI (*n* = 79)	pMCI (*n* = 80)	MCI (*n* = 65) ^1^
tap-cycle_median	−0.593 ± 0.047	−0.433 ± 0.045	−0.440 ± 0.057	−1.442 ± 0.020	−1.380 ± 0.026	−1.365 ± 0.029
tap-cycle_iqr	−2.885 ± 0.081	−2.792 ± 0.080	−2.703 ± 0.099	−3.657 ± 0.066	−3.594 ± 0.050	−3.449 ± 0.080
tap-duration_median	−1.681 ± 0.050	−1.510 ± 0.054	−1.482 ± 0.066	−2.239 ± 0.020	−2.203 ± 0.026	−2.144 ± 0.029
tap-duration_iqr	−3.364 ± 0.074	−3.173 ± 0.081	−3.138 ± 0.089	−3.939 ± 0.046	−3.914 ± 0.037	−3.869 ± 0.053
offphase_median	−1.037 ± 0.051	−0.890 ± 0.049	−0.915 ± 0.060	−2.046 ± 0.025	−1.971 ± 0.032	−1.983 ± 0.035
offphase_iqr	−2.934 ± 0.067	−2.836 ± 0.070	−2.789 ± 0.083	−3.744 ± 0.059	−3.751 ± 0.053	−3.653 ± 0.075
force-peak_median	0.558 ± 0.109	0.527 ± 0.104	0.7901 ± 0.142	0.327 ± 0.089	0.403 ± 0.090	0.725 ± 0.112
force-peak_iqr	−0.449 ± 0.126	−0.594 ± 0.121	−0.328 ± 0.123	−0.454 ± 0.093	−0.420 ± 0.087	−0.189 ± 0.119
time-to-peak_median	−2.397 ± 0.054	−2.246 ± 0.057	−2.195 ± 0.072	−2.979 ± 0.019	−2.940 ± 0.028	−2.884 ± 0.030
time-to-peak_iqr	−3.934 ± 0.095	−3.710 ± 0.111	−3.619 ± 0.112	−4.677 ± 0.045	−4.631 ± 0.039	−4.644 ± 0.057
flexion_median	−3.743 ± 0.082	−3.852 ± 0.081	−3.676 ± 0.107	−3.551 ± 0.080	−3.508 ± 0.074	−3.228 ± 0.096
flexion_iqr	−4.853 ± 0.101	−4.935 ± 0.094	−4.764 ± 0.104	−4.497 ± 0.086	−4.497 ± 0.085	−4.306 ± 0.104
extension_median	−3.735 ± 0.089	−3.839 ± 0.086	−3.628 ± 0.114	−3.650 ± 0.078	−3.601 ± 0.074	−3.324 ± 0.094
extension_iqr	−4.766 ± 0.109	−4.896 ± 0.090	−4.763 ± 0.101	−4.624 ± 0.083	−4.604 ± 0.076	−4.443 ± 0.103
time-to-plateau_median	−2.722 ± 0.038	−2.682 ± 0.040	−2.617 ± 0.050	−3.053 ± 0.015	−3.030 ± 0.021	−2.985 ± 0.021
time-to-plateau_iqr	−4.446 ± 0.100	−4.448 ± 0.089	−4.235 ± 0.108	−4.871 ± 0.041	−4.855 ± 0.042	−4.888 ± 0.057
plateau-duration_median	−2.732 ± 0.076	−2.406 ± 0.084	−2.408 ± 0.097	−3.547 ± 0.025	−3.492 ± 0.032	-3.446 ± 0.040
plateau-duration_iqr	−3.778 ± 0.120	−3.457 ± 0.121	−3.434 ± 0.133	−5.347 ± 0.063	−5.278 ± 0.067	−5.207 ± 0.083

Given are means ± SEM. CHI: cognitively healthy individuals; MCI: participants with mild cognitive impairment; pMCI: participants with possible MCI. Note: units for time values are in log(s), those for force are in log(N) and those for flexion/extension are in log(N/s). Suffix _median specifies group medians and _iqr the inter-quartile range of the group. ^1^: For technical reasons, the data for one participant from the fast pace condition are missing.

**Table 3:** ANOVA results for the self-selected pace condition. Only parameters with significant differences (*p* < 0.05) in at least one effect are shown. *p* values are false-discovery-rate-corrected for multiple comparisons.

Parameter	Effect	DFn	DFd	F	*p*	η^2^_G_
force-peak_median	group	2	219	1.22	0.30	0.01
	sex	1	219	6.32	0.03	0.03
	group × sex	2	219	3.85	0.06	0.03
	FLSD (sex)	0.263				
flexion_median	group	2	219	1.04	0.35	0.01
	sex	1	219	7.43	0.03	0.03
	group × sex	2	219	3.23	0.06	0.03
	FLSD (sex)	0.200				
extension_median	group	2	219	1.18	0.31	0.01
	sex	1	219	6.81	0.03	0.03
	group × sex	2	219	3.36	0.06	0.03
	FLSD (sex)	0.214				
plateau-duration_median	group	2	219	4.49	0.03	0.04
	sex	1	219	1.68	0.30	0.01
	group × sex	2	219	0.99	0.37	0.01
	FLSD (group)	0.238				

DFn: Degree of freedom (nominator); DFd: degree of freedom (denominator); η^2^_G_: generalized effect size; group: CHI, pMCI, MCI; sex: male, female; group × sex: interaction between group and sex; FLSD: Fisher’s least significant difference.

**Table 4:** ANOVA results for the fast pace condition. Only parameters with significant differences (*p* < 0.05) in at least one effect are shown. *p* values are false-discovery-rate-corrected for multiple comparisons.

Parameter	Effect	DFn	DFd	F	*p*	η^2^_G_
tap-cycle_median	group	2	218	3.41	0.03	0.03
	sex	1	218	6.62	0.015	0.03
	group × sex	2	218	5.24	0.015	0.05
	FLSD (group)	0.070				
	FLSD (sex)	0.057				
	FLSD (group × sex)	0.097				
tap-cycle_iqr	group	2	218	3.24	0.06	0.03
	sex	1	218	7.11	0.03	0.03
	group × sex	2	218	2.11	0.12	0.02
	FLSD (sex)	0.147				
tap-duration_median	group	2	218	3.63	0.045	0.03
	sex	1	218	0.10	0.75	0.00
	group × sex	2	218	4.04	0.02	0.04
	FLSD (group)	0.070				
	FLSD (group × sex)	0.098				
offphase_median	group	2	218	2.69	0.07	0.02
	sex	1	218	13.4	<0.001	0.06
	group × sex	2	218	4.51	0.015	0.04
	FLSD (sex)	0.069				
	FLSD (group × sex)	0.117				
offphase_iqr	group	2	218	1.09	0.34	0.01
	sex	1	218	10.8	0.003	0.05
	group × sex	2	218	1.25	0.34	0.01
	FLSD (sex)	0.137				
force-peak_median	group	2	218	4.04	0.02	0.04
	sex	1	218	4.83	0.03	0.02
	group × sex	2	218	1.60	0.20	0.01
	FLSD (group)	0.267				
	FLSD (sex)	0.219				
flexion_median	group	2	218	3.72	0.045	0.03
	sex	1	218	6.34	0.03	0.03
	group × sex	2	218	1.06	0.35	0.01
	FLSD (group)	0.230				
	FLSD (sex)	0.188				
extension_median	group	2	218	3.78	0.03	0.03
	sex	1	218	8.10	0.015	0.04
	group × sex	2	218	1.03	0.36	0.01
	FLSD (group)	0.227				
	FLSD (sex)	0.185				

DFn: Degree of freedom (nominator); DFd: degree of freedom (denominator); η^2^_G_: generalized effect size; group: CHI, pMCI, MCI; sex: male, female; group × sex: interaction between group and sex; FLSD: Fisher’s least significant difference.

**Table 5:** LDA scales.

Parameter	LDA1	LDA2
force-peak_median_self	0.38	0.93
flexion_median_self	−0.61	−0.97
extension_median_self	−0.69	0.21
plateau-duration_median_self	0.50	−0.69
tap-cycle_median_fast	−0.04	−4.50
tap-cycle_iqr_fast	1.01	0.31
tap-duration_median_fast	0.31	2.98
offphase_median_fast	0.17	2.45
offphase_iqr_fast	−1.02	0.09
force-peak_median_fast	−1.42	−3.17
flexion_median_fast	0.91	3.27
extension-median_fast	1.26	−0.15

The suffix _self specifies the parameter of the self-selected pace condition and _fast specifies that of the fast pace condition.

**Table 6:** Confusion matrix of classification.

		Classification Based on Cognitive Assessments		
		CHI	pMCI	MCI
LDA classification	CHI	49	26	18
	pMCI	20	41	24
	MCI	10	13	23

## Data Availability

The datasets analyzed during the current study are not publicly available but are available from the corresponding author on reasonable request.

## Supplementary Materials

The following are available online at https://www.mdpi.com/article/10.3390/jpm12020286/s1, Supplementary Table S1: Mean values of the tapping parameter for both conditions of each group (retransformed into physical dimensions). Supplementary Table S2: Logarithmized tapping parameter of female participants for both conditions and each group. Supplementary Table S3: Logarithmized tapping parameter of male participants for both conditions and each group. Supplementary Figure S1: Histograms of probability densities of LDA values of correctly classified and misclassified participants using only parameters that are significant for the effect group.
