# Supplementary material for: Finger Tapping as a Biomarker to Classify Cognitive Status in 80+-Year-Olds
Source: J Pers Med. 2022 Feb 15;12(2):286. doi: 10.3390/jpm12020286 (PMC8878665; doi:10.3390/jpm12020286)
Supplement: Supplementary file 1 [file jpm-12-00286-s001.zip › jpm-1515036.suppl.pdf]

Journal of *Personalized Medicine*

MDPI

Supplementary Material: Finger Tapping as a Biomarker to Classify Cognitive Status in 80+-Year-Olds

Dieter F. Kutz, Stephanie Fröhlich, Julian Rudisch, Katrin Müller and Claudia Voelcker-Rehage

Table S1. Mean values of the tapping parameters for both conditions of each group (retransformed into physical dimensions).

	Self-Selected Pace			Fast Pace		
	**CHI (*n* = 79)**	**pMCI (*n* = 80)**	**MCI (*n* = 66)**	**CHI (*n* = 79)**	**pMCI (*n* = 80)**	**MCI [1] (*n* = 65)**
tap-cycle_median	0.553	0.648	0.644	0.237	0.252	0.255
tap-cycle_iqr	0.056	0.061	0.067	0.026	0.028	0.032
tap-duration_median	0.186	0.221	0.227	0.107	0.111	0.117
tap-duration_iqr	0.035	0.042	0.043	0.020	0.020	0.021
offphase_median	0.355	0.411	0.401	0.130	0.139	0.138
offphase_iqr	0.053	0.059	0.062	0.024	0.024	0.026
force-peak_median	17.48	16.94	22.04	13.87	14.96	20.65
force-peak_iqr	0.638	0.552	0.721	0.635	0.657	0.834
time-to-peak_median	0.091	0.106	0.111	0.051	0.053	0.056
time-to-peak_iqr	0.020	0.025	0.027	0.009	0.010	0.010
flexion_median	0.024	0.021	0.025	0.029	0.030	0.040
flexion_iqr	0.008	0.007	0.009	0.011	0.011	0.014
extension_median	0.024	0.022	0.027	0.026	0.027	0.036
extension_iqr	0.009	0.008	0.009	0.010	0.010	0.012
time-to-plateau_median	0.066	0.068	0.073	0.047	0.048	0.051
time-to-plateau_iqr	0.012	0.012	0.015	0.008	0.008	0.008
plateau-duration_median	0.065	0.090	0.090	0.029	0.030	0.032
plateau-duration_iqr	0.023	0.032	0.032	0.005	0.005	0.006

Given are group means. CHI: cognitively healthy individuals; MCI: participants with mild cognitive impairment; pMCI: participants with possible MCI. Note: units for time values are in s, those for force are in *n* and those for flexion/extension are in *N*/s. Suffix _median specifies medians and _iqr the inter-quartile range.[1]: For technical reasons, the data for one participant from the fast pace condition are missing.

Table S2. Logarithmized tapping parameters for female participants for both conditions and each group.

	Self-Selected Pace			Fast Pace		
	**CHI (*n* = 44)**	**pMCI (*n* = 37)**	**MCI (*n* = 28)**	**CHI (*n* = 44)**	**pMCI (*n* = 37)**	**MCI (*n* = 28)**
tap-cycle_median	−0.602 ± 0.058	−0.4036 ± 0.071	−0.4914 ± 0.081	−1.390 ± 0.028	−1.303 ± 0.032	−1.406 ± 0.042
tap-cycle_iqr	−2.806 ± 0.099	−2.751 ± 0.124	−2.798 ± 0.145	−3.481 ± 0.088	−3.530 ± 0.071	−3.422 ± 0.100
tap-duration_median	−1.689 ± 0.068	−1.496 ± 0.076	−1.635 ± 0.095	−2.208 ± 0.029	−2.175 ± 0.038	−2.211 ± 0.030
tap-duration_iqr	−3.368 ± 0.111	−3.086 ± 0.125	−3.172 ± 0.149	−3.834 ± 0.051	−3.841 ± 0.052	−3.908 ± 0.080
offphase_median	−1.042 ± 0.059	−0.8404 ± 0.075	−0.9178 ± 0.087	−1.974 ± 0.034	−1.854 ± 0.040	−2.001 ± 0.055
offphase_iqr	−2.885 ± 0.076	−2.786 ± 0.107	−2.824 ± 0.125	−3.578 ± 0.078	−3.653 ± 0.073	−3.590 ± 0.099
force-peak_median	0.4979 ± 0.144	0.4938 ± 0.179	0.2678 ± 0.179	0.2811 ± 0.107	0.3335 ± 0.152	0.4111 ± 0.146
force-peak_iqr	−0.5105 ± 0.157	−0.5945 ± 0.199	−0.6026 ± 0.188	−0.4287 ± 0.107	−0.4882 ± 0.146	−0.3709 ± 0.160
time-to-peak_median	−2.415 ± 0.073	−2.224 ± 0.078	−2.374 ± 0.098	−2.950 ± 0.026	−2.923 ± 0.038	−2.955 ± 0.027
time-to-peak_iqr	−3.978 ± 0.138	−3.626 ± 0.162	−3.710 ± 0.168	−4.590 ± 0.050	−4.608 ± 0.057	−4.683 ± 0.074
flexion_median	−3.797 ± 0.106	−3.906 ± 0.137	−4.066 ± 0.135	−3.616 ± 0.095	−3.591 ± 0.128	−3.486 ± 0.129
flexion_iqr	−4.912 ± 0.120	−4.869 ± 0.161	−5.010 ± 0.143	−4.497 ± 0.101	−4.585 ± 0.147	−4.449 ± 0.150
extension_median	−3.792 ± 0.114	−3.882 ± 0.148	−4.042 ± 0.143	−3.721 ± 0.088	−3.709 ± 0.124	−3.593 ± 0.119
extension_iqr	−4.877 ± 0.128	−4.906 ± 0.147	−5.023 ± 0.138	−4.674 ± 0.092	−4.711 ± 0.132	−4.613 ± 0.134
time-to-plateau_median	−2.709 ± 0.049	−2.658 ± 0.062	−2.720 ± 0.067	−3.023 ± 0.019	−3.023 ± 0.029	−3.032 ± 0.022
time-to-plateau_iqr	−4.508 ± 0.143	−4.429 ± 0.126	−4.307 ± 0.148	−4.776 ± 0.046	−4.841 ± 0.062	−4.892 ± 0.062
plateau-duration_median	−2.781 ± 0.106	−2.397 ± 0.118	−2.598 ± 0.141	−3.527 ± 0.032	−3.454 ± 0.045	−3.539 ± 0.032
plateau-duration_iqr	−3.824 ± 0.181	−3.385 ± 0.170	−3.634 ± 0.197	−5.319 ± 0.081	−5.224 ± 0.110	−5.277 ± 0.103

Given are means ± SEM. CHI: cognitively healthy individuals; MCI: participants with mild cognitive impairment; pMCI: participants with possible MCI. Note: units for time values are in log(s), those for force are in log(N) and those for flexion/extension are in log(N/s). Suffix _median specifies group medians and _iqr the inter-quartile range of the group.

Table S3. Logarithmized tapping parameters for male participants for both conditions and each group.

	Self Selected Pace			Fast Pace		
	**CHI (*n* = 35)**	**pMCI (*n* = 43)**	**MCI (*n* = 38)**	**CHI (*n* = 35)**	**pMCI (*n* = 43)**	**MCI (*n* = 37)**
tap-cycle_median	−0.5813 ± 0.077	−0.4587 ± 0.059	−0.4028 ± 0.079	−1.508 ± 0.026	−1.447 ± 0.038	−1.335 ± 0.040
tap-cycle_iqr	−2.985 ± 0.132	−2.828 ± 0.106	−2.633 ± 0.135	−3.880 ± 0.086	−3.649 ± 0.071	−3.469 ± 0.120
tap-duration_median	−1.672 ± 0.075	−1.523 ± 0.077	−1.370 ± 0.086	−2.278 ± 0.027	−2.227 ± 0.037	−2.092 ± 0.044
tap-duration_iqr	−3.360 ± 0.093	−3.248 ± 0.106	−3.114 ± 0.111	−4.071 ± 0.078	−3.977 ± 0.052	−3.839 ± 0.072
offphase_median	−1.031 ± 0.089	−0.9318 ± 0.064	−0.913 ± 0.084	−2.136 ± 0.033	−2.071 ± 0.045	−1.969 ± 0.046
offphase_iqr	−2.995 ± 0.117	−2.879 ± 0.094	−2.763 ± 0.113	−3.952 ± 0.076	−3.836 ± 0.074	−3.700 ± 0.109
force-peak_median	0.6343 ± 0.167	0.5556 ± 0.119	1.175 ± 0.187	0.3852 ± 0.150	0.4627 ± 0.105	0.9625 ± 0.153
force-peak_iqr	−0.3712 ± 0.207	−0.5939 ± 0.147	−0.1253 ± 0.157	−0.486 ± 0.162	−0.3607 ± 0.102	−0.03877 ± 0.168
time-to-peak_median	−2.373 ± 0.082	−2.265 ± 0.083	−2.062 ± 0.098	−3.015 ± 0.028	−2.954 ± 0.039	−2.830 ± 0.046
time-to-peak_iqr	−3.879 ± 0.127	−3.782 ± 0.154	−3.552 ± 0.151	−4.786 ± 0.077	−4.651 ± 0.054	−4.615 ± 0.083
flexion_median	−3.674 ± 0.130	−3.806 ± 0.094	−3.389 ± 0.141	−3.469 ± 0.134	−3.436 ± 0.084	−3.032 ± 0.129
flexion_iqr	−4.779 ± 0.173	−4.992 ± 0.107	−4.582 ± 0.142	−4.497 ± 0.149	−4.422 ± 0.096	−4.199 ± 0.142
extension_median	−3.664 ± 0.141	−3.802 ± 0.097	−3.323 ± 0.151	−3.561 ± 0.137	−3.508 ± 0.085	−3.121 ± 0.131
extension_iqr	−4.627 ± 0.185	−4.887 ± 0.112	−4.571 ± 0.137	−4.561 ± 0.147	−4.512 ± 0.084	−4.314 ± 0.149
time-to-plateau_median	−2.740 ± 0.061	−2.702 ± 0.051	−2.541 ± 0.070	−3.090 ± 0.022	−3.035 ± 0.031	−2.950 ± 0.032
time-to-plateau_iqr	−4.368 ± 0.137	−4.465 ± 0.127	−4.182 ± 0.153	−4.992 ± 0.069	−4.868 ± 0.057	−4.885 ± 0.090
plateau-duration_median	−2.670 ± 0.110	−2.414 ± 0.121	−2.269 ± 0.129	−3.573 ± 0.039	−3.525 ± 0.045	−3.376 ± 0.063
plateau-duration_iqr	−3.721 ± 0.152	−3.519 ± 0.172	−3.287 ± 0.179	−5.382 ± 0.099	−5.325 ± 0.081	−5.153 ± 0.125

Given are means ± SEM. CHI: cognitively healthy individuals; MCI: participants with mild cognitive impairment; pMCI: participants with possible MCI. Note: units for time values are in log(s), those for force are in log(N) and those for flexion/extension are in log(N/s). Suffix _median specifies group medians and _iqr the inter-quartile range of the group.

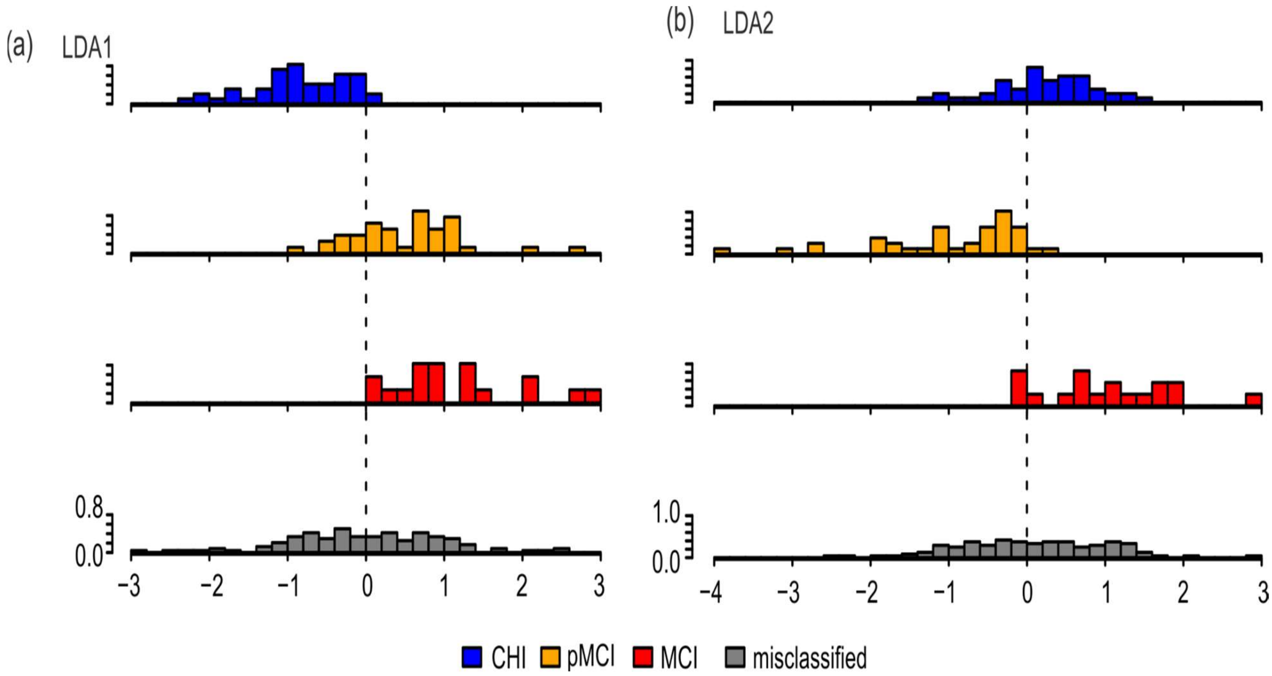

Figure S1. Histograms of probability densities of LDA values of correctly classified and misclassified participants, using only parameters that were significant for the effect group. (**a**) LDA1; (**b**) LDA2. CHI: cognitively healthy individuals; pMCI: participants with possible mild cognitive impairments; MCI: participants with mild cognitive impairments.
